# Detecting Early Signs of Extrapericardial Cardiac Tamponade With ECG

**DOI:** 10.7759/cureus.72590

**Published:** 2024-10-28

**Authors:** Kyungko Huh, Sunao Yamauchi

**Affiliations:** 1 Emergency Medicine, Hiroshima City Hiroshima Citizens Hospital, Hiroshima, JPN; 2 Emergency Medicine, Yuuai Medical Center, Okinawa, JPN

**Keywords:** blunt chest trauma, ecg, extrapericardial cardiac tamponade, retrosternal hematoma, sternal fracture, ventricular ectopy

## Abstract

Cardiac tamponade is a condition with impaired cardiac function by acute fluid accumulation in the pericardium. Extracardiac masses, such as a mediastinal hematoma, can also cause cardiac tamponade. We report a case of impending extrapericardial cardiac tamponade secondary to traumatic sternal fracture with expanding mediastinal hematoma. An 82-year-old male presented to the emergency department after a motor vehicle accident. On arrival, he was hemodynamically stable. An electrocardiogram (ECG) revealed normal sinus rhythm and sporadic premature ventricular complexes (PVCs), which had been documented in his past Holter ECGs. Initial evaluation with a focused sonographic assessment for trauma was negative. However, subsequent computed tomography (CT) showed a small retrosternal hematoma with a sternal fracture and a rib fracture. On day 4, repeated ECG demonstrated more frequent PVCs, a change in morphology, and episodes of non-sustained ventricular tachycardia. Follow-up CT and echocardiogram showed an expanding hematoma and compressed right ventricle. Cardiology and Surgery were consulted, and urgent surgical drainage of the hematoma was performed. The patient was discharged without complications on day 17. An expanding mediastinal hematoma after blunt chest trauma can cause extrapericardial tamponade. In this case, the hematoma caused a change in ECG and increased ventricular ectopy, which served as a critical indicator for successful surgical intervention before circulatory collapse occurred. Telemetry and repeat ECGs should be performed for the first few days following significant chest trauma. This case highlights the utility of continuous ECG monitoring, which provides clinicians with timely recognition of cardiac functional changes and a beneficial indication for appropriate intervention.

## Introduction

Cardiac tamponade is a condition with impaired cardiac function by acute fluid accumulation in the pericardium. Though it is rare, extracardiac masses, such as a mediastinal hematoma, can also cause cardiac tamponade known as extrapericardial cardiac tamponade [1]. Mediastinal hematoma with a sternal fracture is often managed conservatively [2]. On the other hand, the presence of hematoma is considered to be a high risk of cardiac contusion and concomitant injury [3,4]. When the hematoma is large enough or accumulates fast enough to cause extrapericardial cardiac tamponade, hematoma evacuation should be considered. However, the optimal management strategy and follow-up imaging protocol for mild mediastinal hematomas without initial signs of cardiac contusion or pericardial compression remain undefined [5,6]. In addition, the evaluation of hematoma expansion depends on imaging studies. Without imaging, the only possible indicators might be abnormalities in vital signs or a decrease in hemoglobin levels. We report this case to highlight the potential role of ECG changes in the early detection of impending extrapericardial cardiac tamponade, particularly in cases of initially mild mediastinal hematoma.

## Case presentation

**Figure 1:**
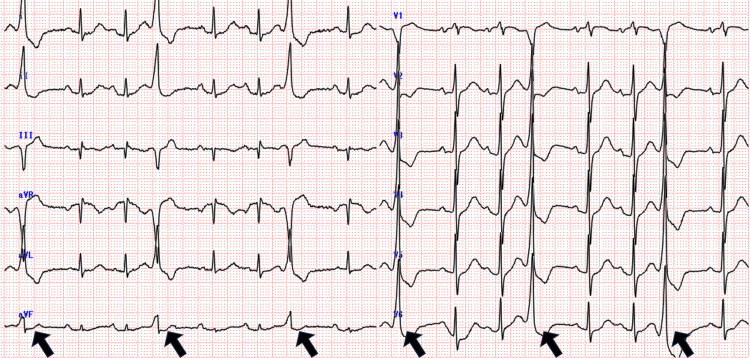
A 12-lead electrocardiogram (ECG) on arrival shows monomorphic premature ventricular complexes (PVCs)

An 82-year-old male was brought to the emergency department (ED) after a motor vehicle accident. On arrival, he was oriented and hemodynamically stable. On physical examination, there was no jugular vein distention. Breath and heart sounds were normal. Left anterior chest wall edema and point tenderness were noted. Initial evaluation with the focused assessment with sonography for trauma (FAST) was negative. An electrocardiogram revealed normal sinus rhythm and sporadic premature ventricular complexes (PVCs), which was the same as previously documented findings in his Holter ECG before the accident (Figure 1).

**Figure 2:**
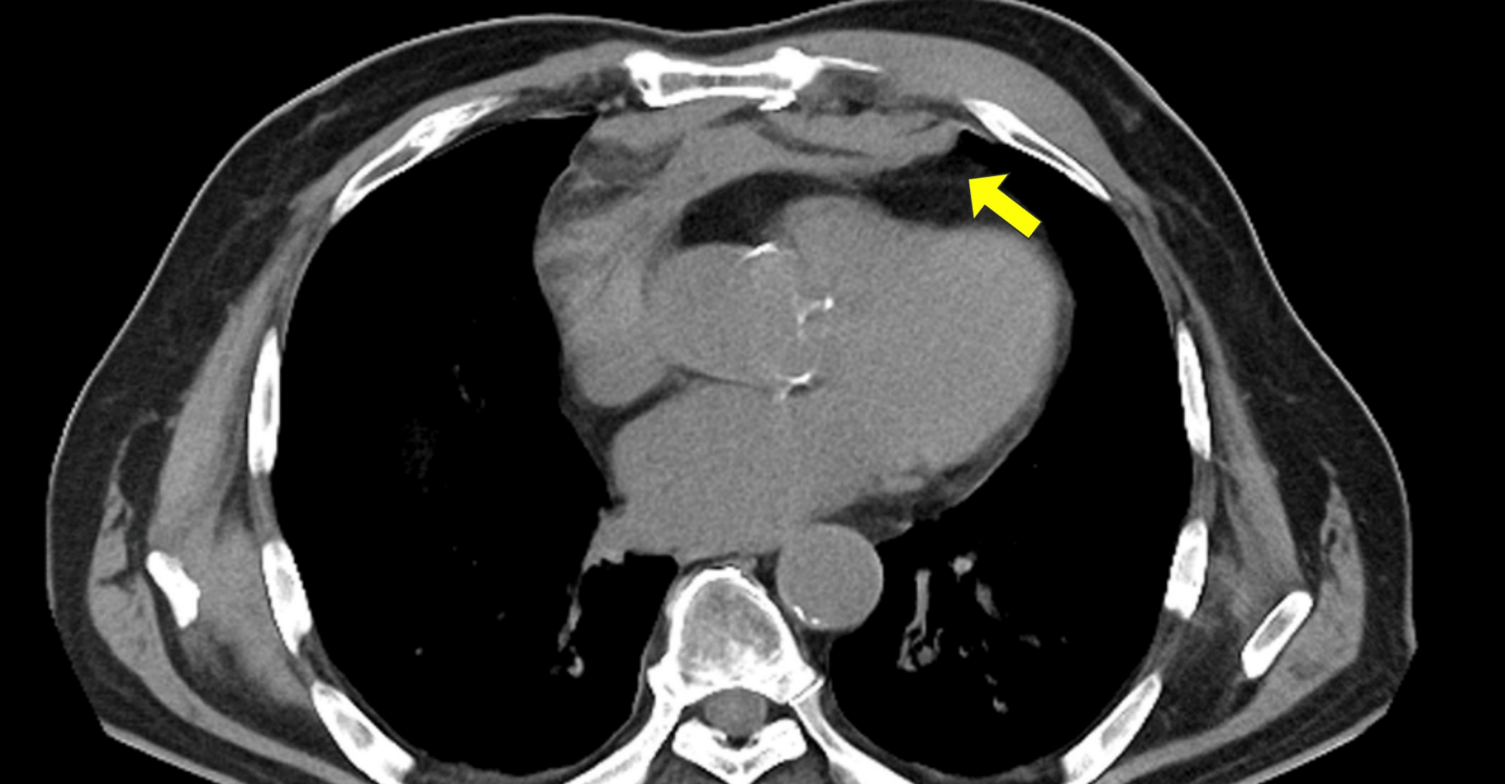
Small mediastinal hematoma, 24 mm in depth from the posterior surface of the sternum

Computed tomography (CT) of the chest revealed multiple rib fractures, a sternal fracture, and a small mediastinal hematoma, 24 mm in depth from the posterior surface of the sternum (Figure 2).

**Figure 3:**
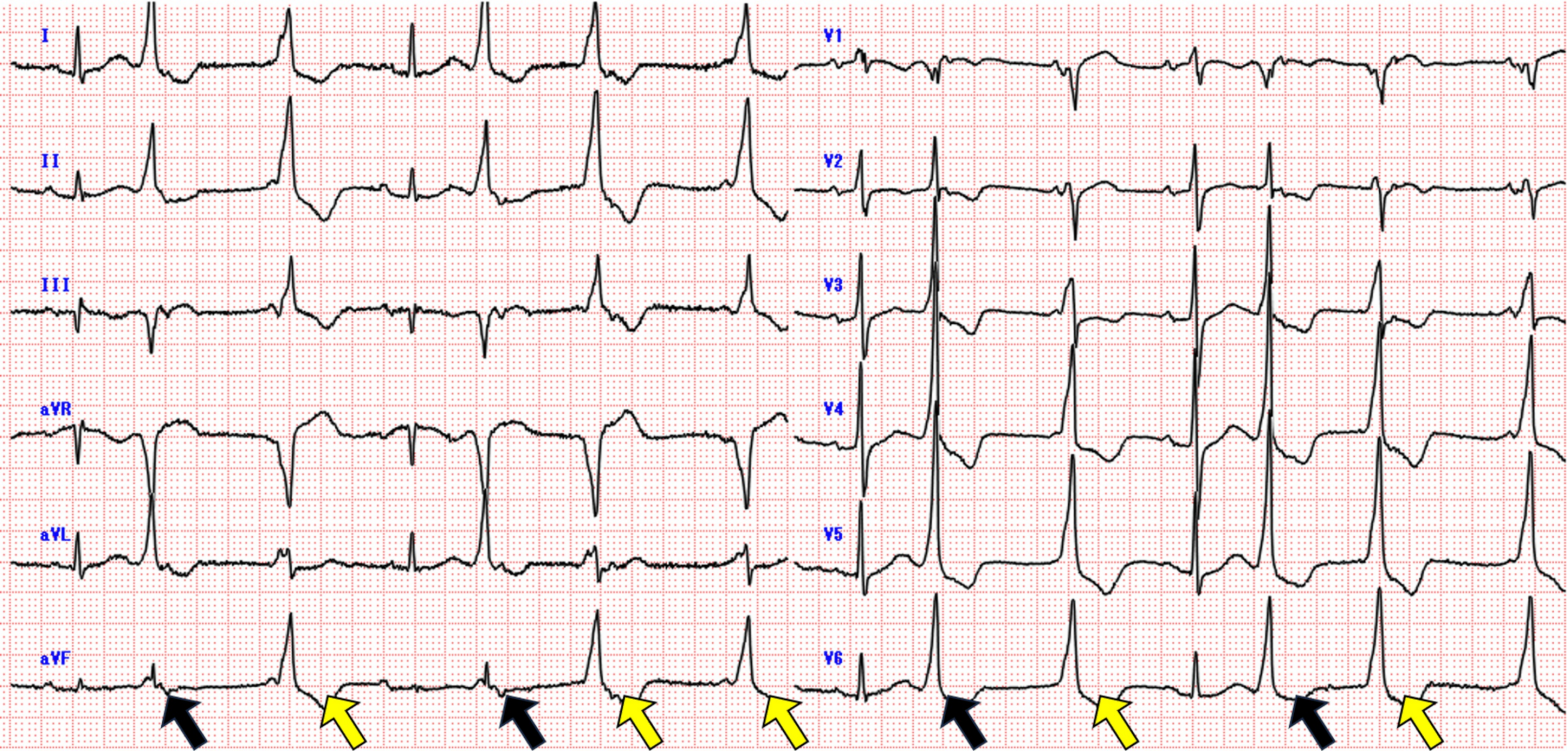
Repeated 12 lead ECG shows a change in morphology of premature ventricular complexes (PVCs) (yellow arrows indicate new-onset PVCs)

Laboratory tests were all within normal ranges. The patient was hemodynamically stable in the ED and hospitalized for observation. On day 2, although the patient’s vital signs remained stable, PVCs became more frequent in telemetry. An echocardiogram revealed an ejection fraction of 68% and a retrosternal hematoma, 26 mm in depth, which was mildly compressing the right chambers. On day 4, repeated ECG demonstrated more frequent PVCs, a change in morphology, and episodes of non-sustained ventricular tachycardia (Figure 3).

**Figure 4:**
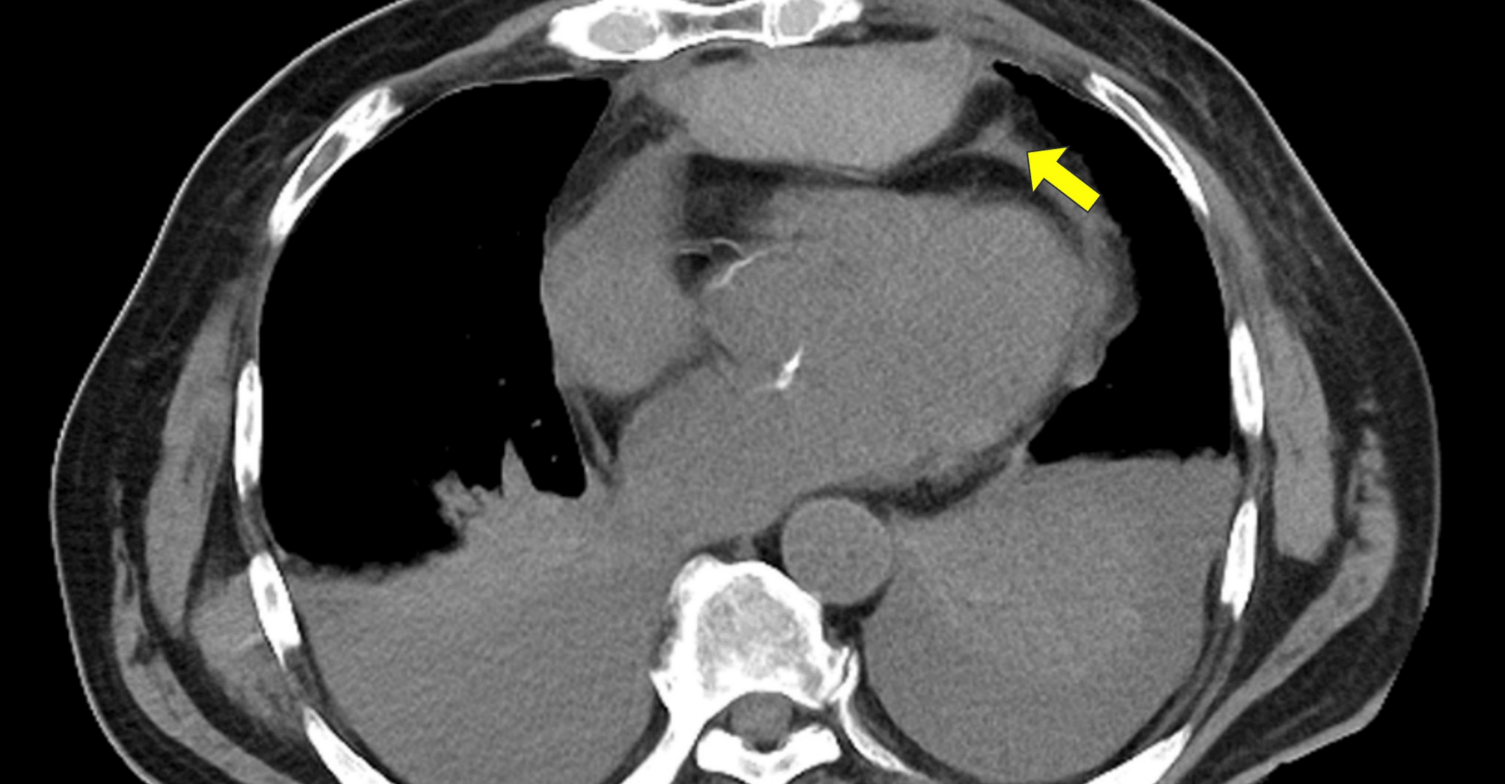
Expanding hematoma, 34 mm in depth from the sternum

A follow-up CT showed a moderately compressed right ventricle by hematoma, 34 mm in depth (Figure 4).

During these events, the patient remained hemodynamically stable. Cardiology and surgery were consulted, and an urgent surgical drainage of hematoma was performed. The source of hemorrhage was a venous oozing from adipose tissue. After the procedure, an ultrasound confirmed normal right ventricular function, and his ventricular ectopy returned to baseline.

## Discussion

We report a case of impending cardiac tamponade due to external compression from a mediastinal hematoma associated with a sternal fracture. In this case, the hematoma was thought to have caused a change in ECG and increased ventricular ectopy. Cardiac tamponade usually results from idiopathic, traumatic, or iatrogenic myocardial or aortic disease, but extrapericardial compression by fluid or solid can also cause cardiac tamponade [1]. Usually, a sternal fracture, even with a small hematoma, is treated supportively and has a good clinical prognosis [2]. However, potential complications to the heart can occur, especially when the fracture is accompanied by mediastinal hematoma. Misdiagnosis and delay of treatment may result in lethal outcomes for patients [7].

Hemodynamic instability due to cardiac tamponade is related to how rapidly the hematoma spreads out rather than the size of the hematoma [8]. In the more common pericardial tamponade, rapid accumulation of even a small amount (50-100 ml) of fluid can cause cardiac compromise. With chronic accumulation, the pericardium is capable of accommodating large amounts as the pericardial compliance increases over time [9,10]. In our case, venous hemorrhage with slow increase provided cardiac chambers enough time to develop compliance and likely avoided hemodynamic collapse. The mediastinum is significantly larger than the pericardial space and can handle more fluid/mass, which is also considered to contribute to slow deterioration in this case. However, Rami et al. reported a case of mediastinal hematoma that immediately deteriorated and resulted in fatality [7]. Clinicians should keep a high suspicion for extrapericardial cardiac tamponade even if the mediastinal hematoma is initially minimal.

Echocardiography is a gold standard test to identify cardiac tamponade. Our initial FAST exam did not demonstrate an abnormal cardiac function, but the subsequent CT showed a small mediastinal hematoma. It is possible that the mediastinal hematoma was overlooked with the standard FAST protocol. Hsu et al. recommend extended FAST with a parasternal long-axis view to minimize the risk of missing insidious mediastinal hematoma [11]. CT can be considered another option to identify a mediastinal hematoma, which has higher sensitivity to confirm minimal changes [7,11].

The ECG remains a crucial tool for investigating cardiac injury in blunt chest trauma, with repeated ECGs being essential for monitoring the progression of such injuries [6]. However, for diagnosing cardiac tamponade, echocardiography and CT are generally preferred over ECG, as they provide more reliable guidance for both diagnosis and therapeutic intervention [12]. Extrapericardial cardiac tamponade presents a unique challenge, as it encompasses both cardiac injury and partial external compression. In our case, the presence of PVCs with varying morphologies served as a critical indicator for surgical intervention, ultimately leading to successful treatment before circulatory collapse occurred. We believe that continuous ECG monitoring and telemetry can play a vital role in identifying progressive cardiac dysfunction resulting from mediastinal hematoma. While the clinical significance of PVCs may be limited, the development of PVCs in extrapericardial cardiac tamponade could serve as an important factor in the early detection of cardiac compression due to mediastinal hematoma and surgical decision-making.

## Conclusions

An expanding mediastinal hematoma after blunt chest trauma can cause extrapericardial tamponade. Even if the hematoma is minimal and subclinical at first, it can progress over time to be large enough to compromise cardiac function. While CT remains the gold standard for diagnosing a mediastinal hematoma, ECG and telemetry monitoring may enable the early detection of extrapericardial tamponade without imaging studies. Therefore, the development of PVCs and other arrhythmias in this context may provide crucial information for determining the timing of surgical intervention.
